# Supplementary figures and images for: Positive Feedback and Noise Activate the Stringent Response Regulator Rel in Mycobacteria
Source: PLoS One. 2008 Mar 12;3(3):e1771. doi: 10.1371/journal.pone.0001771 (PMC2258413; doi:10.1371/journal.pone.0001771)

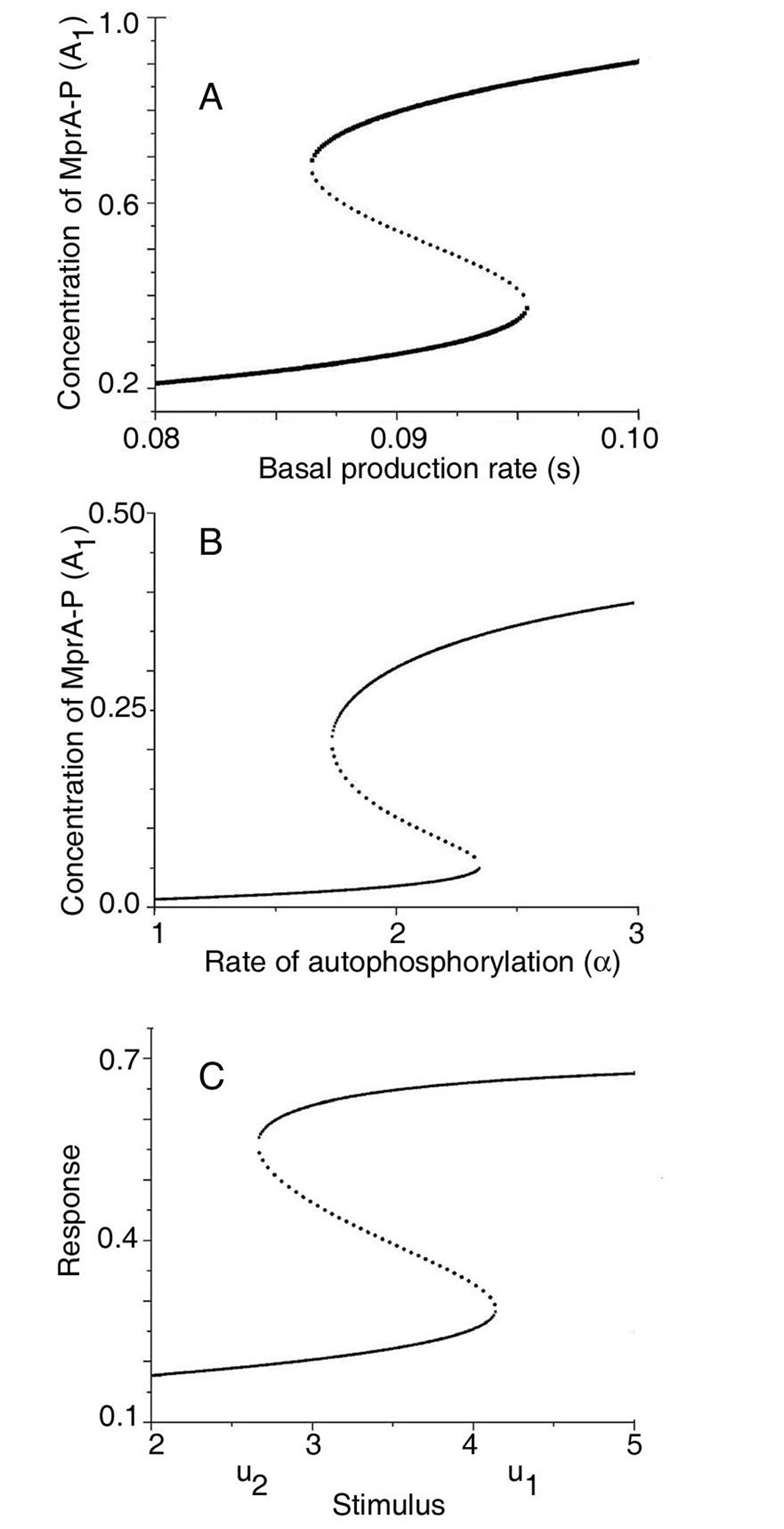

Supplement: Figure S1 — Bistability and hysteresis in the deterministic model. Steady state concentrations of phosphorylated MprA versus basal production rate s (Eq. 2) (A) and the parameter α (Eq. 19) (B). A generic stimulus-response curve in the steady state. The points u1 and u2 denote the upper and lower bifurcation points respectively at which transitions occur from one branch to the other. (6.62 MB TIF) [file pone.0001771.s001.tif]

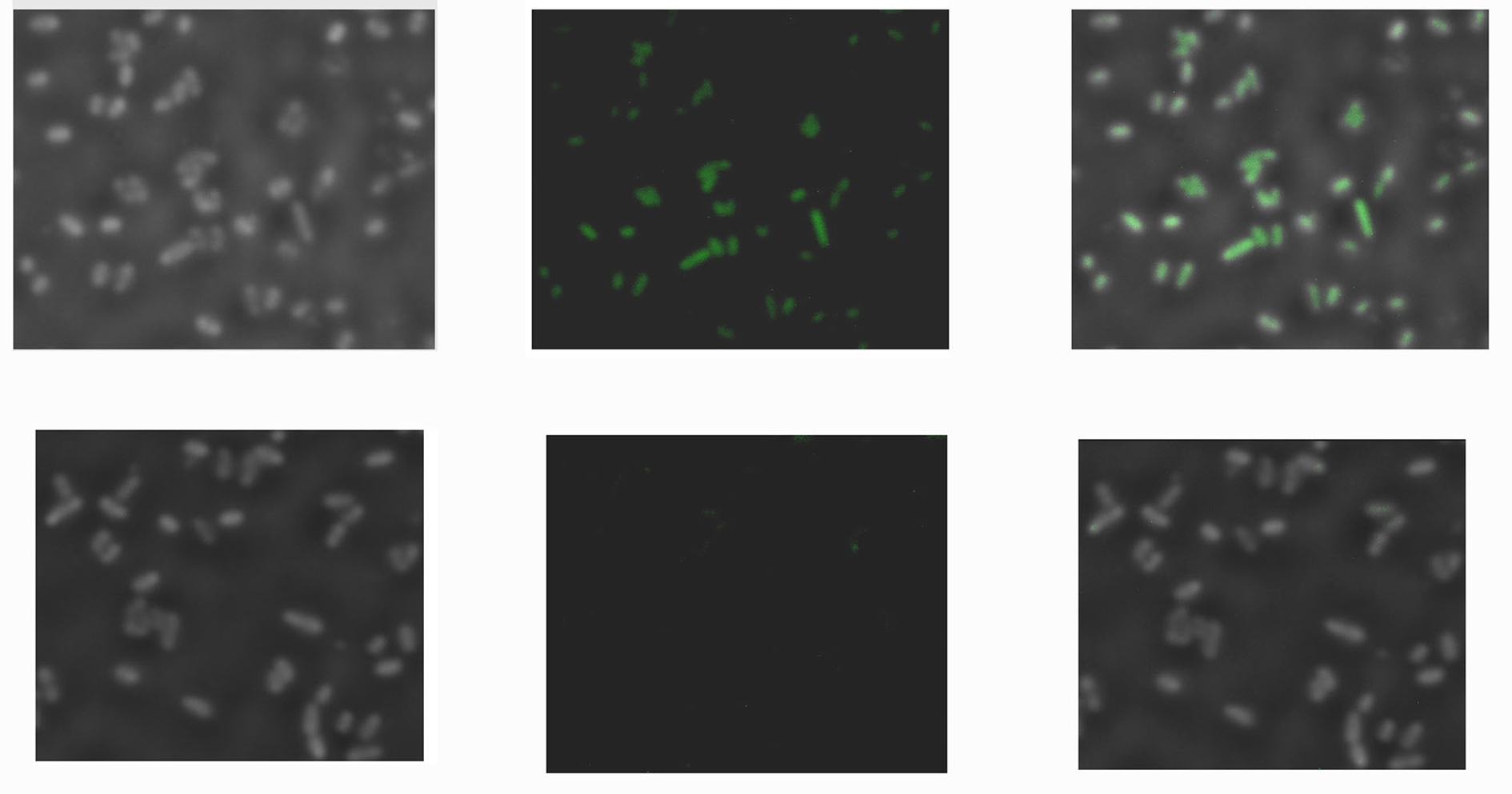

Supplement: Figure S2 — Fluorescence microscopy of rel-GFP expression. M. smegmatis harboring rel-GFP was grown as described in Fig. 2 and expression of GFP was visualized by fluorescence microscopy. Left, middle and right micrographs : phase contrast, fluorescence and merge respectively. Top panel: Rel-High (44 h); Bottom panel: Rel-Low (12 h) (5.64 MB TIF) [file pone.0001771.s002.tif]

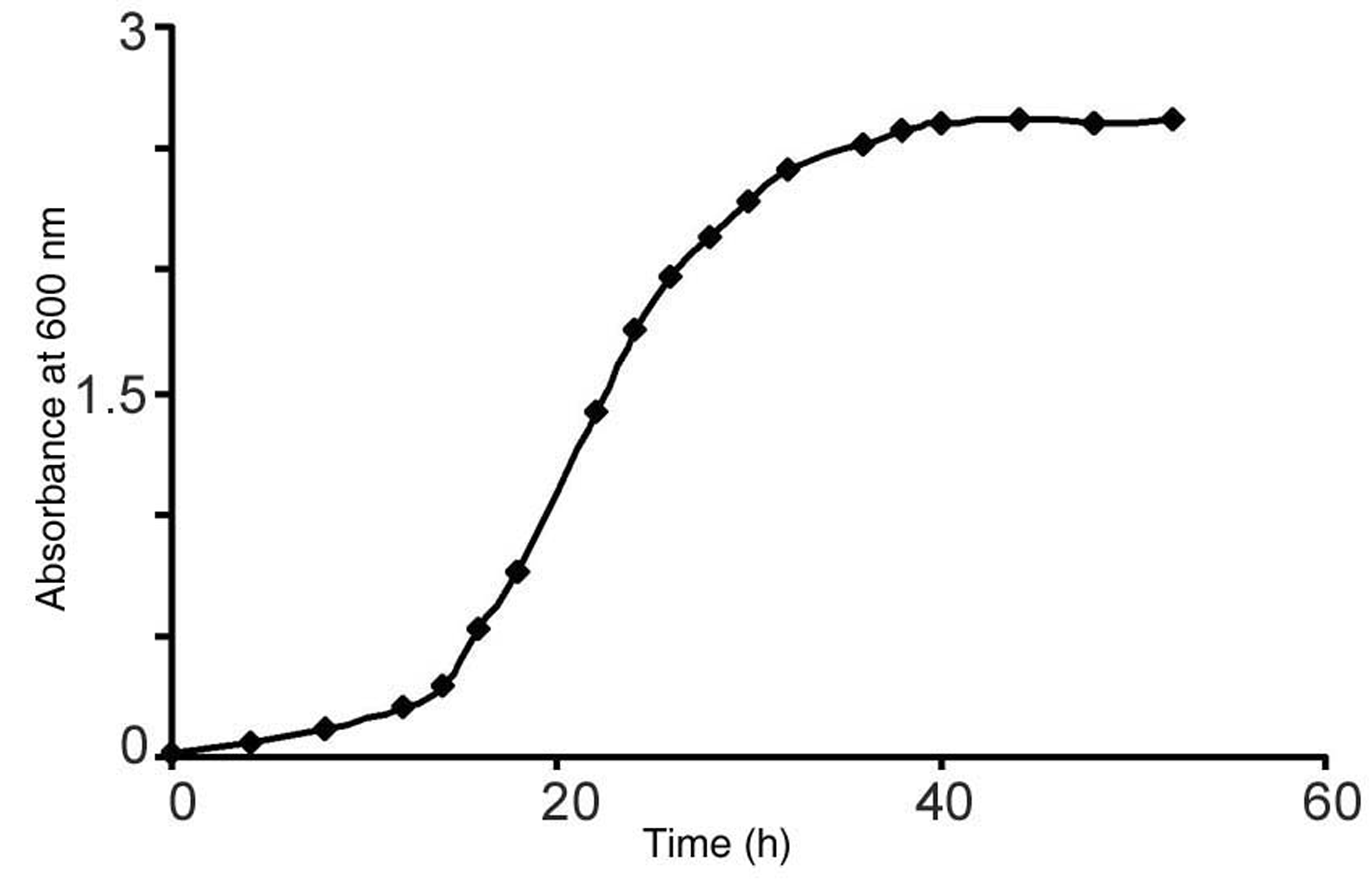

Supplement: Figure S3 — Growth kinetics of M. smegmatis mc2155. Cells were grown for different periods of time as indicated and the growth was monitored by recording the absorbance at 600 nm spectrophotometrically. (2.94 MB TIF) [file pone.0001771.s003.tif]

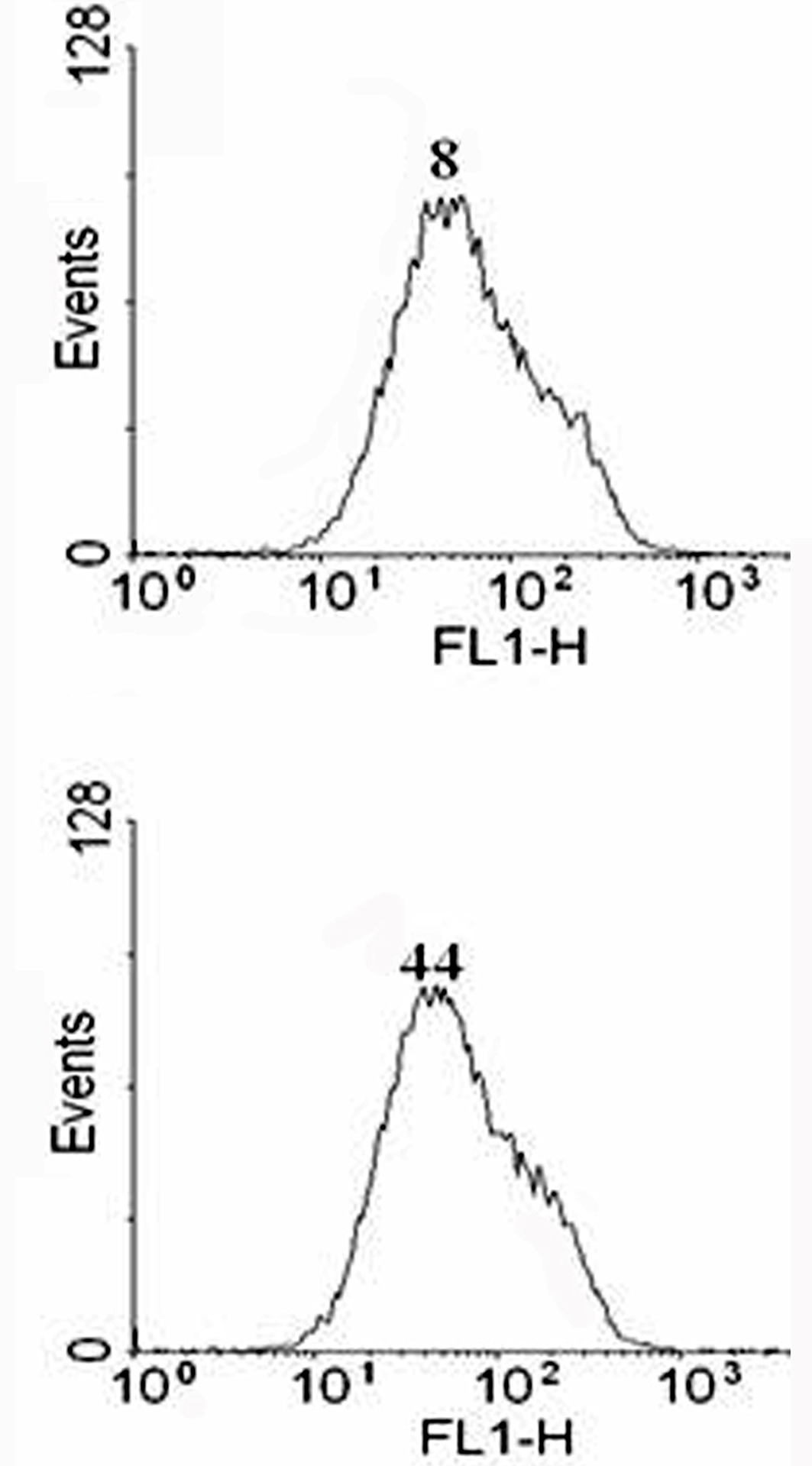

Supplement: Figure S4 — Time course of hsp-GFP expression. M. smegmatis harboring hsp-GFP was grown for different periods of time and the expression of GFP was monitored by flow cytometry as described in Fig. 2. Representative histograms obtained after 8 and 44 h of growth are shown above. At all time points, a single peak (as shown above) was observed. (6.13 MB TIF) [file pone.0001771.s004.tif]

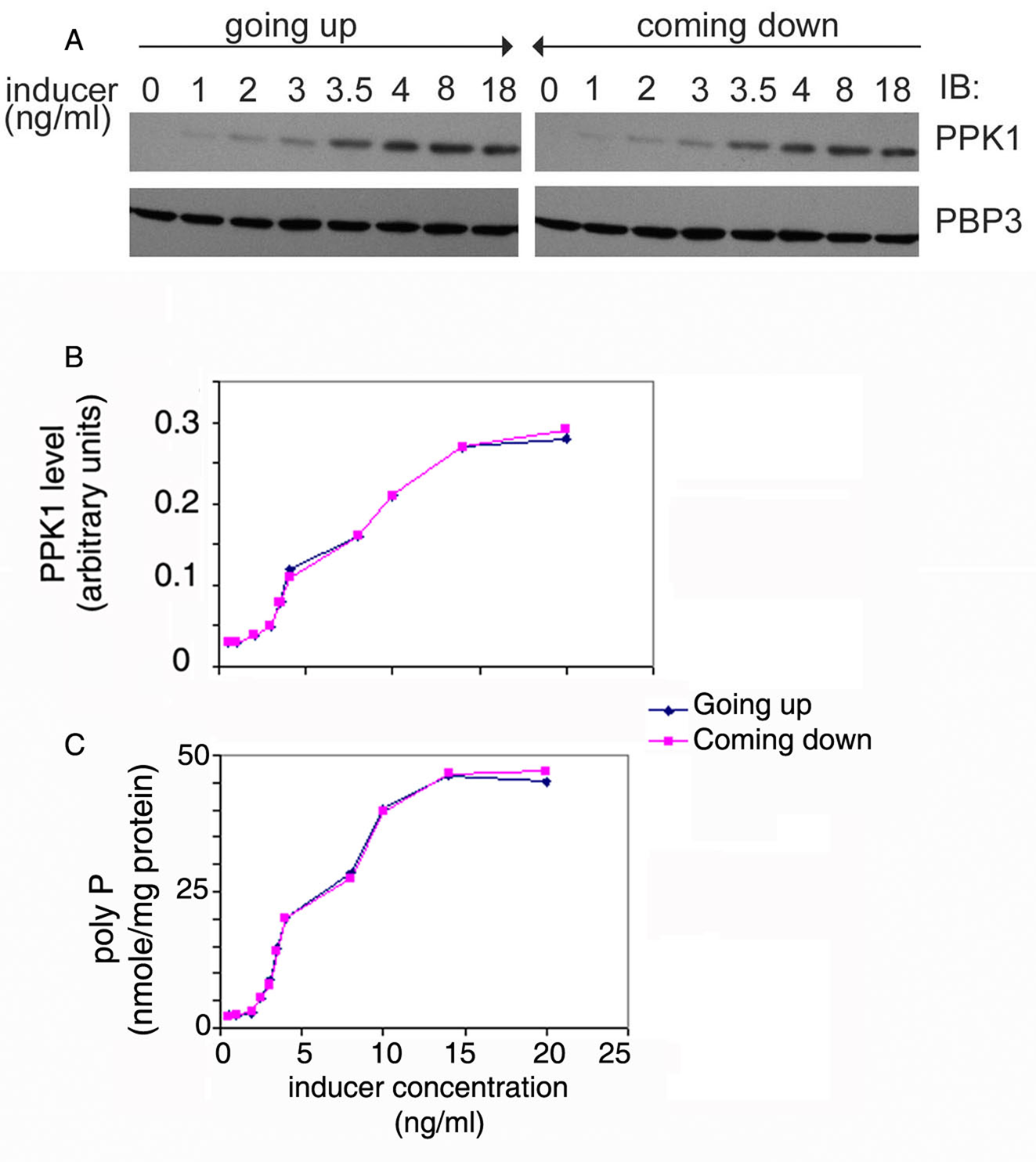

Supplement: Figure S5 — A. Western blots of tetracycline-inducible PPK1 in PPK-KO. PPK-KO harboring the integrated tet-inducible ppk1 and the construct of rel-gfp was grown upto stationary phase in the presence of different concentrations of tetracycline (inducer). Lysates were analyzed by SDS-PAGE and Western blotting using anti-PPK1. Blots were reprobed with anti-PBP3 to ensure equal loading. B. Densitometric analysis of Western blots (panel A) of PPK1. C. Cells were grown as described under panel A, and poly P levels were measured as described in Ref. 1. (5.90 MB TIF) [file pone.0001771.s005.tif]

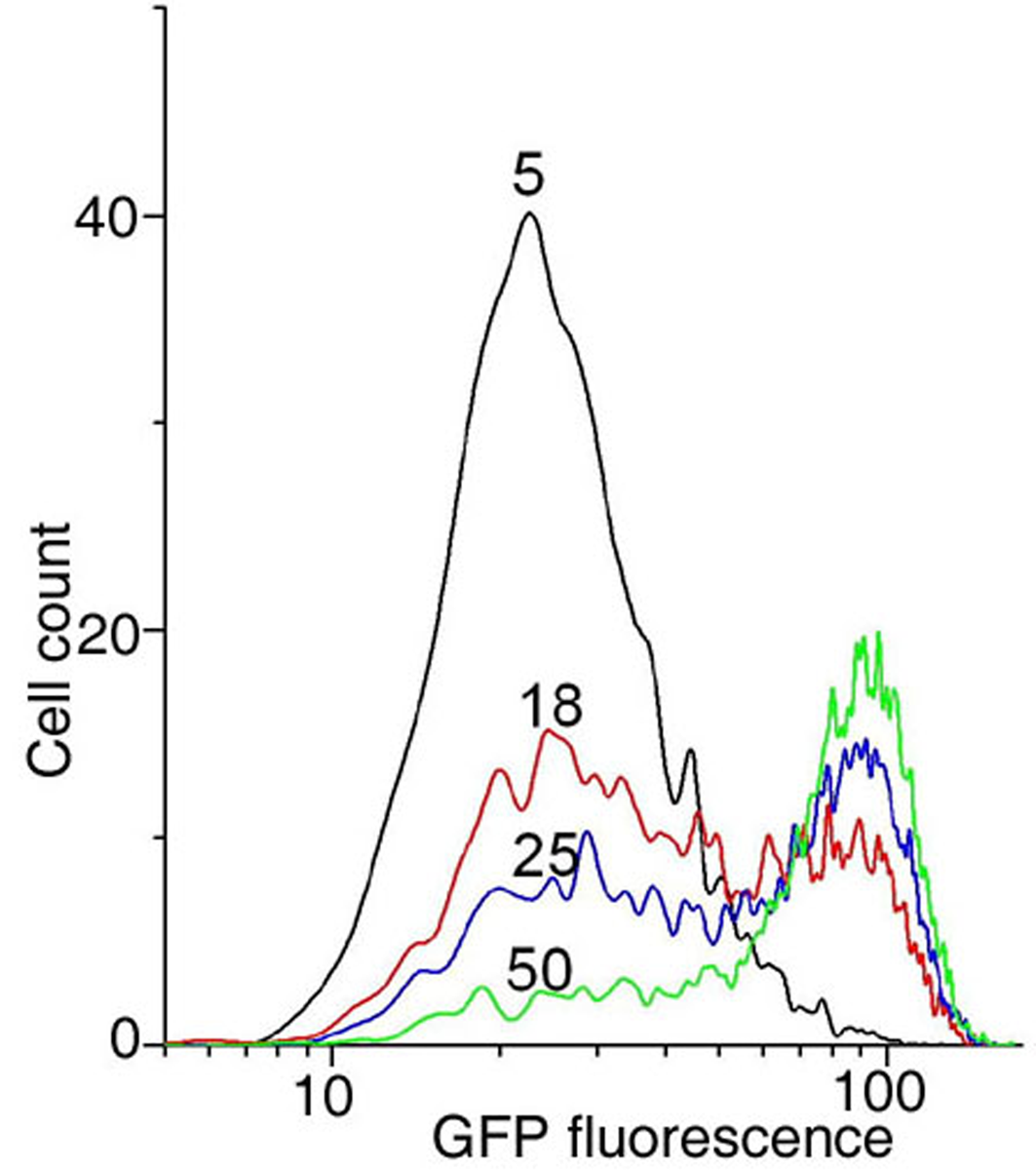

Supplement: Figure S6 — GFP distributions obtained through stochastic simulation via the Gillespie algorithm. 5, 18, 25 and 50 represent time in h. (3.84 MB TIF) [file pone.0001771.s006.tif]

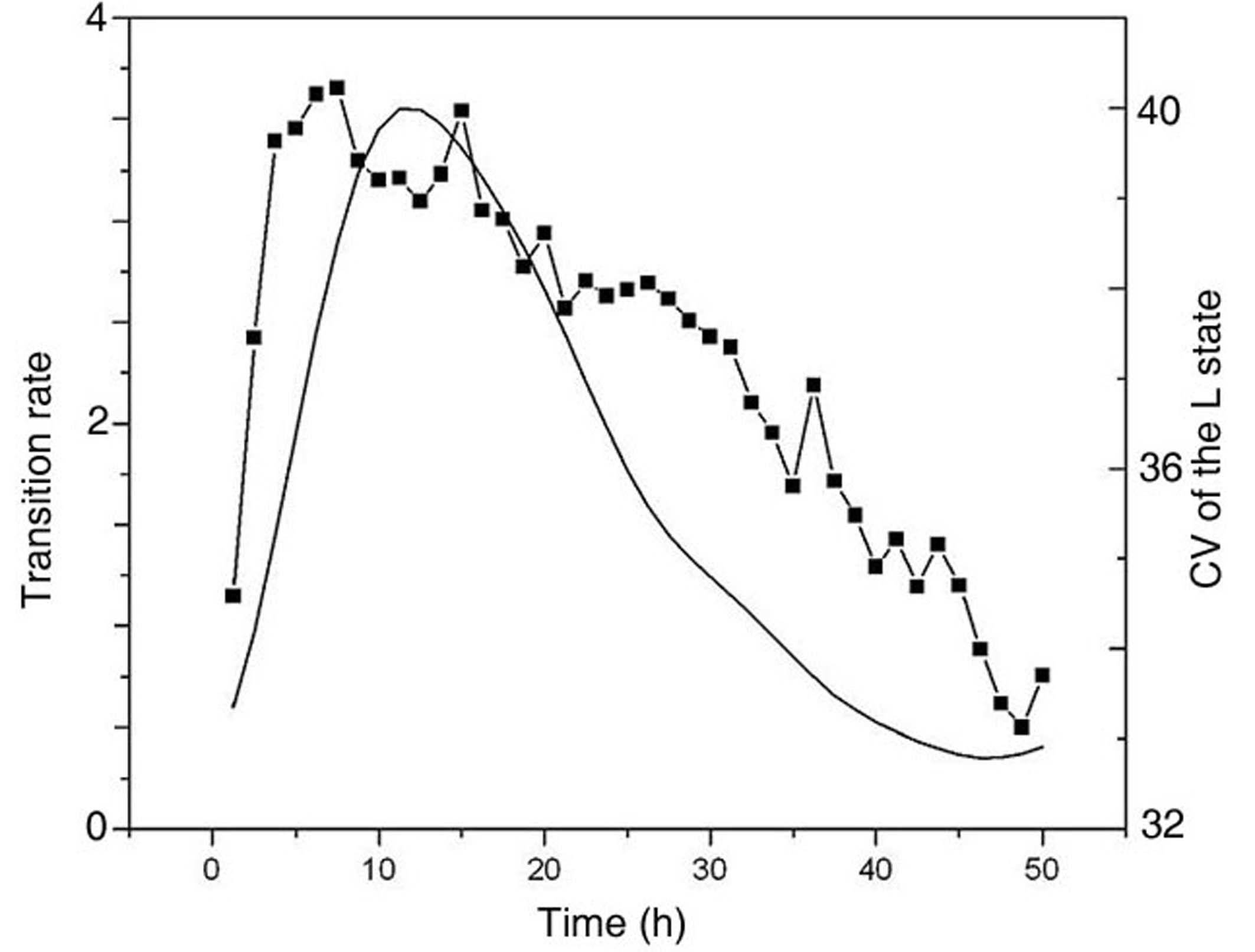

Supplement: Figure S7 — Rate of transition from the L to the H state and the CV of the low expression state versus time obtained through stochastic simulation based on the Gillespie algorithm. (3.44 MB TIF) [file pone.0001771.s007.tif]

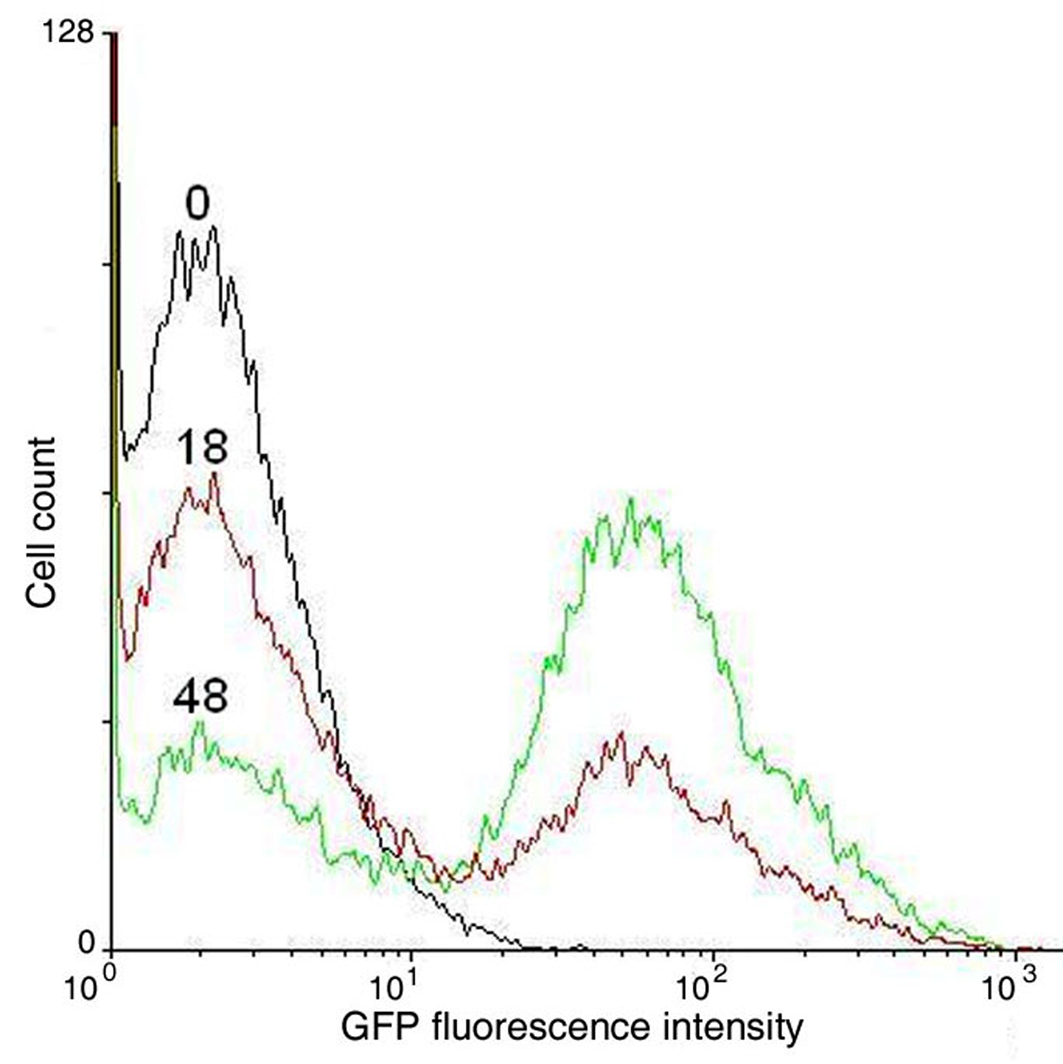

Supplement: Figure S8 — Time course of sigE-GFP expression. M. smegmatis expressing the sigE-GFP construct was grown for different periods of time (0, 18, 48 h) as indicated and the expression of GFP was monitored by flow cytometry. The figure provides evidence of bimodal sigE expression. (3.40 MB TIF) [file pone.0001771.s008.tif]
