# Supplementary material for: Positive Feedback and Noise Activate the Stringent Response Regulator Rel in Mycobacteria
Source: PLoS One. 2008 Mar 12;3(3):e1771. doi: 10.1371/journal.pone.0001771 (PMC2258413; doi:10.1371/journal.pone.0001771)
Supplement: Text S1 — Mathematical models (0.14 MB DOC) [file pone.0001771.s009.doc]

**Mathematical models**

Deterministic Model

The reaction scheme describing the events shown in Fig 1 is given by

(1)

(2)

(3)

(4)

(5)

In the equations, A_1_ (A_2_) denotes the phosphorylated (unphosphorylated) form of MprA and B_1_ (B_2_) denotes the phosphorylated (unphosphorylated) form of MprB. G_AB_ and G_AB_^*^ represent the inactive and active states of the *mprAB* operon respectively. In the inactive state, MprA and MprB are synthesized at a basal rate s and in the active state protein production occurs at an enhanced rate β. Eq. (1) denotes the autophosphorylation reaction of MprB with the phosphate groups taken from the poly P chain. Eq. (2) denotes the transfer of the phosphate group from phosphorylated MprB to MprA. Eq. (3) corresponds to dephosphorylation of phosphorylated MprA by phosphorylated MprB which thus acts as a phosphatase. Eqs. (4) and (5) describe activation of the *mprAB* operon by phosphorylated MprA and basal expression of the operon respectively. Refs. 1-3 provide experimental justification for the reaction scheme given in Eqs. (1)-(5). Using standard mass action kinetics, we write down the rate equations for the concentration of each of the molecular species participating in the biochemical events. The equations are:

(6) (7)

(8)

(9)

(10)

(11)

(12)

(13)

(14)

Eq. (13) represents SigE synthesis due to transcriptional activation of the *sigE* gene by phosphorylated MprA-P. Eq. (14) describes GFP production due to the activation of the *rel* promoter by SigE. The rate constants γ, δ_1_ and δ_2_ are the decay constants, each having two components: the protein degradation rate constant and the rate constant associated with dilution due to cell growth.

The signaling pathway in Fig 1 is a dynamical system the time evolution of which is described by Eqs. (6)-(14). In the limit of long time, the system reaches a steady state in which the concentrations of the participating molecules attain fixed values. The steady state solution is obtained by setting all the rates of change to be zero. In the case of bistability, three steady state solutions are possible: two stable and one unstable. To illustrate the origin of bistability in a simple manner, we assume γ=δ_1_=δ_2_. In the steady state, one has to solve a set of four coupled nonlinear algebraic equations:

(15)

(16)

(17) (18)

where

$\alpha=\frac{k_{1} k_{3}}{k_{2}+k_{3}}, \alpha_{1}=\frac{k_{4} k_{6}}{k_{5}+k_{6}}, \alpha_{2}=\frac{k_{7} k_{9}}{k_{8}+k_{9}}, k=\frac{k_{d}}{k_{a}}$ (19)

The solutions of equations (15)-(19) are obtained with the help of Mathematica. Fig S1A exhibits the steady state concentrations of phosphorylated MprA as a function of the basal production rate s. The different parameters have values

(20)

Fig S1B shows the solutions generated by varying the parameter α (associated with the autophosphorylation of MprB). The parameter $s$ has the value $s$= 0.07. The other parameters have values as in Eq. (20). In Figs S1A and S1B, the regions of bistability can be identified by the existence of three steady state solutions corresponding to the same parameter values. The solid and dotted branches represent stable and unstable steady states respectively.The conclusions of the theoretical study remain valid over a wide parameter range. To obtain experimental confirmation of bistable response, we designed an experiment in which the parameter α could be varied. For this purpose, the *ppk1* gene was placed under the control of the inducible *tet* promoter and integrated into the *ppk1*-inactivated strain. We used the inducer tetracycline to control the level of PPK1 and therefore the synthesis of the poly P chain. Since the latter acts as the source of phosphate groups for the autophosphorylation of MprB (Eq. 1), the rate constant $k_{1}$ in equation (1) is effectively proportional to the inducer (or the PPK1) concentration. Figure S1C shows the generic inducer-response curve which includes a region of bistability. In this case, the same amount of stimulus (inducer) can evoke two different types of response, namely, low and high gene expression levels. The region of bistability separates two regions of monostability. The nature of the response (low or high) in the bistable region is history-dependent, i.e., depends on initial conditions [4]. If the inducer concentration is initially low (high), the system is in the low “L” (high “H”) response state. As the inducer concentration is changed, cells continue to remain in the L (H) state till the threshold value $u_{1} \left(u_{2}\right)$ is reached. At $u_{1} \left(u_{2}\right)$, a discontinuous jump to the H (L) state occurs and the system becomes monostable. Bistability is accompanied by hysteresis, i.e., the amount of stimulus required for the switch from the L to the H state is greater than the amount to bring about the reverse transition. The two stable branches are separated by a branch of unstable steady states (dotted line) which are not experimentally accessible. A well-known analogy is that of two valleys (stable steady states) separated by a hill (unstable steady state corresponds to the hill top). Examples of systems in which hysteresis have been observed experimentally are few in number[5-9] bistable systems, hysteretic response is often masked due to rapid stochastic transitions between the low and high expression states. This is the case of destabilized memory in which the dynamical behavior of the system is history-independent [10]. Systems exhibiting hysteresis have a persistent memory with the two steady states serving as the memory states. In the mathematical model, the expression due to the activation of the *rel* promoter by SigA is included in the basal production rate $s_{2}$ of GFP (Eq. (14)).

Stochastic Model

The reactions shown in Eqs. (1)-(5) as well as SigE and GFP production and degradation are probabilistic in nature giving rise to fluctuations (noise) around mean protein levels. The gene expression noise is responsible for the switching transitions from the L to the H state. There is a critical amount $x_{c}$ of proteins which separates the L and H states. When protein levels are below (above) $x_{c}$, the system attains the L (H) state in the course of time. In the deterministic scenario, since an unstable steady state separates the two stable steady states, $x_{c}$ corresponds to the protein concentration in the unstable steady state. When stochasticity is taken into account, $x_{c}$ itself becomes a fluctuating quantity. A L to H transition takes place when the basal level crosses $x_{c}$. Fluctuations in the basal level promote the chances of such crossings. In the hill-valley analogy, noise enables the system to cross the barrier from one stable steady state to another. The reverse transition, H to L, is less probable due to the robustness associated with hysteresis.

We carried out stochastic simulation based on the Gillespie algorithm [11] for the reaction scheme shown in Fig. 1. We took into consideration the fact that the reactions shown in Eqs. (1)-(3), specially the formation of the intermediate complexes, occur on a faster time scale than the gene expression processes in Eqs. (4)-(5). Thus the first three reactions can be approximated as effective one step processes with the intermediate complexes attaining their steady states. In the case of second order reactions, the deterministic reaction rates must be divided by $N_{A} V$ ($N_{A}$ is the Avogadro number and V the cell volume) to convert the concentrations into numbers of molecules. The reactions considered in the stochastic formalism are

(21)

(22)

(23)

(24)

(25)

(26)

(27)

(28)

(29)

(30)

The rate constants $\alpha, \alpha_{1}$ and $\alpha_{2}$ are as defined in Eq. (19). The parameter values used for the simulation are

The rate constant $k_{1}$ in $\alpha$ (Eq.(19)) is taken to be proportional to the tetracycline inducer concentration $(p)$ and $\alpha$ is fixed as $\alpha=0.5 p$. Fig. S6 shows the GFP distributions as a function of time. The bimodal character of the distribution becomes more prominent as time progresses reaching the steady state in the stationary phase. Fig. S7 shows the variation of the rate of transition from the L to the H state and the coefficient of variation (CV) associated with the basal expression level, as a function of time. The plots in Figs. S4 and S5 reproduce the qualitative features of the experimental plots (Figs. 2 and 3C). The simulation has been carried out for a population of 5000 cells.

**References**

1 Sureka K, Dey S, Datta P, Singh AK, Dasgupta A, et al. (2007) Polyphosphate kinase is involved in stress-induced *mprAB-sigE-rel* signaling in mycobacteria. Mol Microbiol 65: 261-276.

2 Zahrt TC, Wozniak C, Jones D, Trevett A (2003) Functional analysis of the *Mycobacterium tuberculosis* MprAB two-component signal transduction system. Infect Immun 71: 6962-6870.

3 He H, Zahrt TC (2005) Identification and characterization of a regulatory sequence recognized by *Mycobacterium tuberculosis* persistence regulator MprA. J Bacteriol 187: 202-212.

4 Ferrell JE Jr. (2002) Self-perpetuating states in signal transduction: positive feedback, double-negative feedback and bistability. Curr. Opin. Cell Biol. 14: 140-148.

5 Gardner TS, Cantor CR, Collins JJ. (2000) Construction of a genetic toggle switch in *Escherichia coli.* Nature 403: 339-342.

6 Sha W, Moore J, Chen K, Lassaletta AP, Yi CS, et al. (2003) Hysteresis drives cell-cycle transitions in Xenopus laevis egg extracts. Proc. Natl. Acad. Sci. USA 100: 975-980.

7 Pomerening JR, Sontag ED, Ferrell JE Jr. (2003) Building a cell cycle oscillator: hysteresis and bistability in the activation of Cdc2. Nature Cell Biol **5:** 346-351.

8 Ozbudak EM, Thattai M, Lim HN, Shraiman, BI, van Oudenaarden A (2004) Multistability in the lactose utilization network of *Escherichia coli.* Nature 427: 737-740.

9 Kramer BP, Fussenegger M (2005) Transgene control engineering in mammalian cells. Proc Natl Acad Sci USA 102: 9517-9522.

10 Acar M, Beckskei A, van Oudenaarden A (2005) Enhancement of cellular memory by reducing stochastic transitions. Nature 435: 228-232.

11 Gillespie DT (1977) Exact stochastic simulation of coupled chemical reactions. *J. Phys.* Chem 81: 2340-2361.
