# Supplementary material for: Positive Feedback and Noise Activate the Stringent Response Regulator Rel in Mycobacteria
Source: PLoS One. 2008 Mar 12;3(3):e1771. doi: 10.1371/journal.pone.0001771 (PMC2258413; doi:10.1371/journal.pone.0001771)
Supplement: Table S1 — Primers used for mutation of the rel promoter (0.02 MB DOC) [file pone.0001771.s010.doc]

Table S1

Primers used for mutations of the *rel* promoter

________________________________________________________________________

Primer b Primer c Mutation

________________________________________________________________________

5'-TCTGAGGGAGAGCCTCGAATTCGG -3' 5'-CCGAATTCGAGGCTCTCCCTCAGA -3' TATCCT→GAGCCT

(SigA site)

5'-ATCGCCGGGCGCAAGATCATCGACG-3' 5'-CGTCGATGATCTTGCGCCCGGCGA-3' deletion of the -10

binding site (CGTTG)

of SigE

Primer a

5'-TAA**GGTACC**GAAGTTGTCGAAATGACGT-3' [ KpnI site in bold]

Primer d

5'- TA**GGATCC**TGGTTGTCACCTCCTGCCCA-3' [ BamHI site in bold]

______________________________________________________________________________________
